# Osteonectin bidirectionally regulates osteoblast mineralization

**DOI:** 10.1186/s13018-023-04250-1

**Published:** 2023-10-08

**Authors:** Yun‑Sen Zhu, Ting‑Ting Mo, Chang Jiang, Jiang‑Nan Zhang

**Affiliations:** Department of Orthopaedic Surgery, The First People’s Hospital of Wenling, Chuan’an Nan Road NO 333, Wenling, 317500 Zhejiang China

**Keywords:** Osteonectin, p38 MAPK signaling pathway, Discoidin domain receptor 2, Mineralization

## Abstract

**Objective:**

The aim of this study was to investigate whether Osteonectin/Secreted protein acidic and rich in cysteine (ON/SPARC) had a two-way dose-dependent regulatory effect on osteoblast mineralization and its molecular mechanism.

**Methods:**

Initially, different concentrations of ON were added in osteoblasts, and the gene of bone sialoprotein (BSP), osteocalcin (OCN), osteopontin (OPN) and alkaline phosphatase (ALP) were detected using reverse‐transcription quantitative polymerase chain reaction (RT‐PCR). Secondly, based on the above results, the Optima and inhibitory concentration of ON for osteoblast mineralization were determined and regrouped, the Control group was also set up, and the gene detections of Collagen 1 (Col 1), Discoidin domain receptor 2 (DDR2) and p38 mitogen‑activated protein kinase were added using RT‐PCR. In the third stage of the experiment, osteoblasts were pretreated with 0.4Mm ethyl-3,4-dihydroxybenzoate (DHB) (a specific inhibitor of collagen synthesis) for 3 h before adding the optima SPARC, the gene and protein expressions of OCN, OPN, BSP, ALP, DDR2, ALP, Col 1, DDR2 and P38 were detected by RT‑qPCR and western blot analysis, and the mineralized nodules were observed by alizarin red staining.

**Results:**

The results showed that the expression of OCN, OPN, BSP, ALP, DDR2, ALP, Col 1, DDR2 and P38 genes and proteins in osteoblasts were significantly enhanced by 1 ug/ml ON, 100 ug/ml ON or 1 ug/ml ON added with 3,4 DHB significantly inhibited the expressions of DDR2, P38 and the above-mentioned mineralization indexes, and significantly reduced the formation of mineralized nodules.

**Conclusion:**

This study suggested that ON had a bidirectional dose-dependent regulatory effect on osteoblast mineralization, and the activation of P38 pathway by collagen binding to DDR2 was also an important molecular mechanism.

## Introduction

Extracellular matrix (ECM) mineralization is a key step in bone repair and remodeling. As an important non-collagen protein, Osteonectin/Secreted protein acidic and rich in cysteine (ON/ SPARC) plays a critical role in the initiation and regulation of mineralization. ON regulates the synthesis of ECM and collagen [1, 2], affects the differentiation, maturation and mineralization of osteoblasts [3–6], and has high affinity for type 1 collagen and hydroxylapatite (HA) [7]. Its importance to osteoblast mineralization has also been widely demonstrated in gene knockout animal experiments [8, 9]. Studies of biomimetic mineralization have also provided the same evidences that modeling of SPARC structural regions contributes to efficient apatite deposition [10] and that nanocomposite bound to SPARC effectively induce and permit the ordered growth of crystals in bone [11]. ON is thought to be involved in the entire process of extracellular matrix mineralization.

Non-collagen proteins regulate the composition of organic and mineralized substances, regulate cell activity and interactions with the cell matrix, which is the structural basis of maintaining the elasticity and mechanical resistance of mature tissues [12]. Non-collagen protein plays a central role in bone formation and bone turnover, and its bidirectional regulation ensures the normal process of mineralization [13]. The transfer and mineralization of minerals on the surface of collagen fibers are closely related to the observed presence of non-collagen proteins, and these family members may act as accelerators or suppressors, its specific role depends on the concentration factor and temperature [14]. Similar studies have shown that ON has a negative effect on osteoblast mineralization. Several in vitro experiments have shown that ON can inhibit HA formation [15, 16], and confirmed that at high concentrations, it shows alack of nucleating activity [13]. However, there have been few such experimental studies.

Our previous studies have demonstrated that 1 ug/ml of ON has a significant positive regulatory effect on osteoblast mineralization [17], and the activation of p38 mitogen‑activated protein kinase (MAPK) pathway by Recombinant Discoidin Domain receptor 2 (DDR2) is its important molecular mechanism [18]. The aim of this study was to investigate whether ON had a two-way dose-dependent regulatory effect on osteoblast mineralization and its molecular mechanism.

## Materials and methods

### Culture and identification of osteoblasts

Osteoblasts were purchased from Abace Biotechnology company, washed with phosphate‐buffered saline for two times, and suspended in α‐minimum essential medium (*α*‐MEM). After identified by alkaline phosphatase (ALP) staining and alizarin red staining (ARS), osteoblasts were cultured for subsequent experiments.

### Cell grouping and induced mineralization

The second-generation osteoblasts were re-inoculated in a six‐well plate at a density of 2 × 10^5^ cells/mL; they were then cultured in α‐MEM with 10% FBS. After mixing the cells, the original medium was aspirated, and the cells were randomly assigned to different groups and studied in three stages.

### Stage one

A (Control) group, adding mineralized solution (containing 50 mg/L ascorbic acid, 10 mmol/L sodium glycerophosphate, and 100 ml/L FBS α‐MEM culture solution); B group, adding mineralized solution + 1 μg/mL SPARC (SINO Biological 80,870‐R08H); C group, adding mineralized solution + 10 μg/mL SPARC; D group, adding mineralized solution + 100 μg/mL SPARC; and E group, adding mineralized solution + 500 μg/mL SPARC. The medium in each group was substituted every 2 days. The gene expressions of non-collagen proteins (BSP, bone sialoprotein; OCN, osteocalcin; and OPN, osteopontin) and ALP were detected by reverse‐transcription quantitative polymerase chain reaction (RT‐PCR) on the 5th day (according to the previous study [17]). The inhibitory SPARC concentration was determined based on the above results.

### Stage two

Control group, the osteoblasts were added with mineralized solution; Optima group, the osteoblasts were added with mineralized solution + 1 µg/mL ON; Inhibitory group, the osteoblasts were added with mineralized solution + inhibitory SPARC. The medium was substituted every 2 days in each group. On day 5, the non-collagen protein (OPN, BSP, OCN), Col1 a1, Col1 a2, Ddr2 and P38 gene expressions were detected using RT-qPCR. The experiments were conducted in triplicates.

### Stage three

Control group, the osteoblasts were added with mineralized solution; Optima group, the osteoblasts were added with mineralized solution + 1 µg/mL ON; Inhibitor1 group (inhibitory SPARC concentration group), the osteoblasts were added with mineralized solution + inhibitory SPARC; Inhibitor2 group, the osteoblasts were added with mineralized solution + 1 µg/mL ON + 0.4 Mm ethyl-3,4-dihydroxybenzoate (DHB) (in this group, osteoblast was pretreated with 3,4-DHB for 3 h before adding the optima SPARC and mineralized solution). The medium was substituted every 2 days in each group. On day 5, the Col1 a1, Col1 a2, Ddr2, p38, OPN, BSP, OCN and ALP gene and protein expressions were determined using RT-PCR and Western Blot. The experiments were conducted in triplicates. The mineralized nodules were stained using ARS.

### RT-qPCR

Cells were collected from each group, and the total ribonucleic acid (RNA) was extracted using a Trizol reagent (No. 15596026; Ambion Company) in accordance with the manufacturer’s protocols. Next, 2 µg of total RNA was used for reverse transcription, and an RT‐PCR was performed using an RT‐PCR kit (No. RR037A; Takara Company) in accordance with the manufacturer’s instructions. The Col1 a1, Col1 a 2, Ddr2, P38, ALP, OPN, BSP, and OCN gene expression levels were analyzed using the Light Cycler® 96 real‐time PCR system (Roche), with glyceraldehyde 3-phosphate dehydrogenase (GAPDH) as an internal control gene. The primer sequences are listed in Table 1. All real‐time PCRs were performed in triplicate, and the results after calibration with GAPDH expression were calculated using the ΔΔCT method; they are presented in fold increase and relative to the control.

**Table 1:** Primer sequences for RT‐qPCR

Target gene	Primer sequence (5′– 3′)
Mouse COL1 a1	F: GACGCCATCAAGGTCTACTG
	R: ACGGGAATCCATCGGTCA
Mouse COL1 a2	F: GGAGGGAACGGTCCACGAT
	R: GAGTCCGCGTATCCACAA
Mouse DDR2	F: CTCCCAGAATTTGCTCCAG
	R: GCCACATCTTTTCCTGAGA
Mouse P38	F: GGATATTTGGTCCGTGGGCT
	R: CCGTCAGACGCATTATCTGC
Mouse OPN	F: CCAGCCAAGGACCAACTACA
	R: AGTGTTTGCTGTAATGCGCC
Mouse BSP	F: AGAAAGAGCAGCACGGTTGA
	R: AATCCTGACCCTCGTAGCCT
Mouse OCN	F: ATTGTGACGAGCTAGCGGAC
	R: TCGAGTCCTGGAGAGTAGCC
Mouse ALP	F: GATGTGGAGTATGAGAGTGACG
	R: GGTCAAGGGTCAGGAGTTC
Mouse GAPDH	F: CCTGCACCACCAACTGCTTA
	R: CATCACGCCACAGCTTTCCA

COL1: Collagen 1; DDR2: Disk protein domain receptor 2; P38: P38 mitogen‐activated protein kinase; BSP: Bone sialoprotein; OCN: Osteocalcin; OPN: osteopontin; ALP, alkaline phosphatase; F: Forward; R: Reverse; RT‐qPCR: Reverse transcription quantitative polymerase chain reaction

### Western blot analysis

The proteins extracted from osteoblasts were quantified using a bicinchoninic acid assay protein assay kit (Beyotime) in accordance with the manufacturer’s instructions. The cells cultured in the six-well plate were washed with PBS three times, and an appropriate amount of RIPA lysate (Beyotime) was added to phenylmethylsulfonyl fluoride (PMSF) (Amersco-0754-100G) within a few minutes before use (the sinal PMSF concentration was 1 mM). Then, 200 µL of pyrolysate was added to each hole and mixed well; after full decomposition and 10,000 × g centrifuging for 5 min, the supernatant was taken. A suitable amount of BCA working fluid (P0011, Beyotime) was prepared by adding 50 volumes of BCA reagent A and 1 volume of BCA reagent B (50:1), the appropriate volume of the sample was added to a 1.5 mL centrifuge tube and supplemented with a 0.9% NaCL solution to 100 µL. Next, 1 mL of BCA working fluid was added to each hole and left at 37 °C for 30 min; the A562 absorption value was then determined and the protein concentration calculated according to the standard curve. The protein of each group was loaded on sodium dodecyl sulfate‐polyacrylamide gel electrophoresis and transferred onto nitrocellulose membranes. The membranes were blocked with 5% skim milk in Tris-buffered saline with Tween 20 at room temperature for 1 h. They were then supplemented with the primary antibody (1:1000) and incubated at 4 °C for 12 h. The membranes were then incubated with a secondary antibody (1:2000) at room temperature for 2 h. Protein bands were developed using enhanced chemiluminescence reagents (Millipore), the gel gray scales were captured using the ImageJ (V1.8.0) software, and the relative expression quantity was calculated using the grayscale-to-GAPDH ratio.

### ALP staining

The original culture medium was removed, and the cells were washed twice using PBS and fixed in 2.5% glutaraldehyde for 24 h. They were then washed three to five times with PBS and stained with an ALP solution (BCIP-NBT, C3206, Beyotime) in the dark for 30 min. After removing the BCIP-NBT dyeing solution, the cells were washed in distilled water two to three times and observed under a microscope.

### ARS

A volume of 1% alizarin red aqueous solution was obtained by dissolving 1 g of alizarin red powder (A5533, Sigma) in 100 mL of distilled water. Impurities were filtered, the PH adjusted to 4.2 with 10% ammonia, and the solution stored at 4 °C for later use. After a two-week mineralized solution induction, the osteoblasts were washed with PBS and fixed with 95% ethanol for 30 min. After drying, the prepared ARS solution was added for 15 min; the osteoblasts were then rinsed three times with distilled water, dewatered, sealed, and observed and photographed under a light microscope (Olympus) for calcification detection. ImageJ was used to calculate the image-field percentage of positive calcium nodule staining.

### Statistical analysis

All data were tested for normality and homogeneity of variance; the measurement data were presented with the mean ± standard deviation (SD). A one-way analysis of variance was conducted for the comparison among multiple groups, and tests on the least significant difference (Student–Newman–Keuls test or q test) were conducted for the comparison between two groups with homogeneity of variance; comparisons between two groups without homogeneity of variance were highlighted using Tamhane’s T2 test. A *P* value of < 0.05 was considered statistically significant. All statistical analyses were performed using the SPSS (17.0) software.

## Results

### 100 μg/ml SPARC had significant negative effects on the expression of osteoblast mineralization genes

In the first stage of the experiment, the osteoblasts were divided into five groups. On day 5, RT‐qPCR was conducted to measure the mRNA expressions of BSP、OCN、OPN and ALP.

**Fig. 1:**
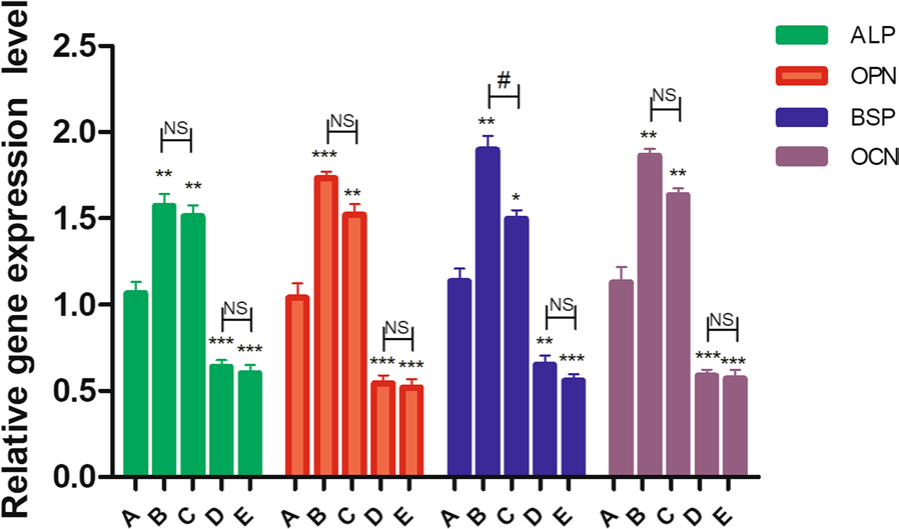
Different concentrations of ON had different effects on the expression of osteoblast mineralization genes. the mRNA expressions of BSP、OCN、OPN and ALP were quantified by RT‐qPCR; *P < .05, **P < .01, ***P < .001 versus the A group; #*P* < .05, ##*P* < .01, ###*P* < .001 Comparison between Group B and C/ Group D and E; the experiments were conducted in triplicates; data were expressed by means ± standard deviation (SD). ON/SPARC, osteonectin/secreted protein acidic and rich incysteine; ALP, alkaline phosphatase; BSP, bone sialoprotein; OCN, osteocalcin; OPN, osteopontin; RT‐qPCR: reverse transfection quantitative polymerase chain reaction; Group A: adding mineralized solution; Group B: adding mineralized solution + 1 μg/ml SPARC; Group C: adding mineralized solution + 10 μg/ml SPARC; Group D: adding mineralized solution + 100 μg/ml SPARC; Group E: adding mineralized solution + 500 μg/ml SPARC

The results showed that, compared with Group A, the BSP, OCN, OPN and ALP expressions were significantly increased in the group B and C (Group B: ALP, *P* < 0.01; OPN, *P* < 0.001; BSP, *P* < 0.01; OCN, *P* < 0.01. Group C: ALP, *P* < 0.01; OPN, *P* < 0.01; BSP, *P* < 0.05; OCN, *P* < 0.01), while significantly inhibited in group D and E (Group D: ALP, *P* < 0.001; OPN, *P* < 0.001; BSP, *P* < 0.01; OCN, *P* < 0.001. Group E: ALP, *P* < 0.001; OPN, *P* < 0.001; BSP, *P* < 0.001; OCN, *P* < 0.001). There was no significant difference between Group B and Group C (ALP/OPN/OCN, *P *> 0.05; BSP, *P* < 0.05), and between Group D and Group E(ALP/OPN/OCN/BSP, *P *> 0.05) (Fig. 1).

Based on the above results, 1 μg/ml SPARC was selected as the Optima concentration and 100 μg/ml SPARC as the inhibitory concentration.

### The inhibitory concentration of ON reduced the gene expression of Ddr2 and P38

In the second stage of the experiment, RT‐qPCR was conducted to measure the mRNA expressions of Col1 a1, Col1 a2, Ddr2 and P38.

**Fig. 2:**
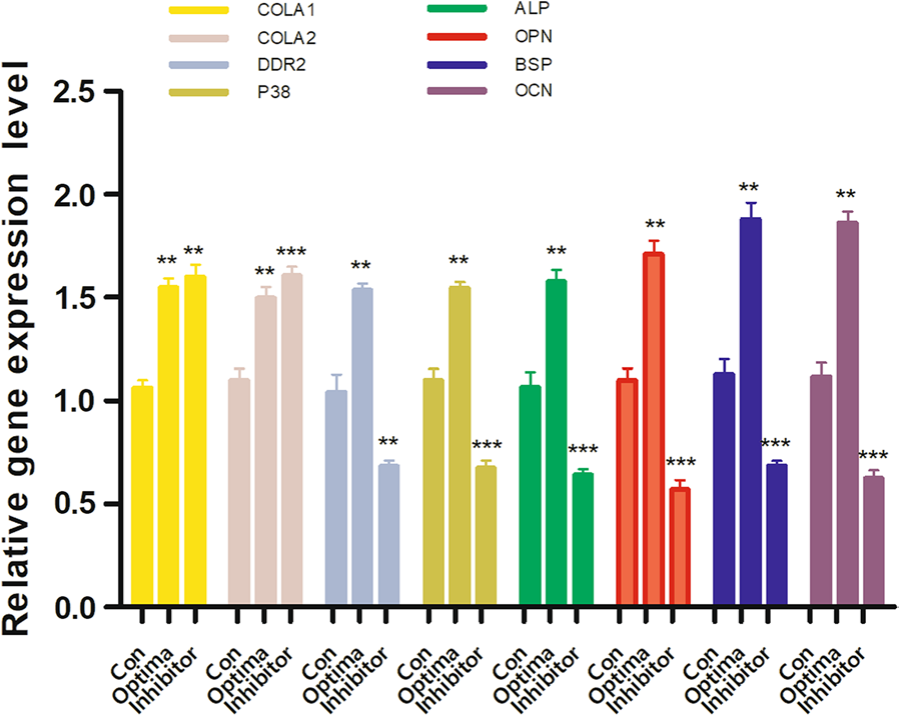
The inhibitory concentration ON reduced the gene expression of DDR2 and P38. the mRNA expressions of t Col1 a1, Col1 a2, DDR2 P38、BSP、OCN、OPN and ALP were quantified by RT‐qPCR; *P < .05, **P < .01, ***P < .001 versus the Con group; the experiments were conducted in triplicates; data were expressed by means ± standard deviation (SD). ON/SPARC, osteonectin/secreted protein acidic and rich incysteine; COL1: collagen 1; DDR2: the disk protein domain receptor 2; P38: p38 mitogen‐activated protein kinase; ALP, alkaline phosphatase; BSP, bone sialoprotein; OCN, osteocalcin; OPN, osteopontin; RT‐qPCR: reverse transfection quantitative polymerase chain reaction; CON group: adding mineralized solution; Optima group: adding mineralized solution + 1ug/ml SPARC; Inhibitory group: adding mineralized solution + 100ug/ml SPARC

Compared with the Control group, the gene expression of Ddr2 and P38 increased significantly in the optimal group (Ddr2/P38, *P* < 0.01) and decreased significantly in the inhibitory group (Ddr2, *P* < 0.01; P38, *P* < 0.001), while the Col1 a1, Col1 a2 gene expression increased obviously in both groups (Group Optima: Col1 a1/ a2, *P* < 0.01. Group Inhibitory: Col1 a1, *P* < 0.01; Col1 a2, *P* < 0.001) (Fig. 2).

### The inhibitor of collagen synthesis and the inhibitory concentration of ON have obvious negative regulatory effect on osteoblast mineralization

In the third stage of the experiment, 3,4-DHB was added into the Optima group, 3,4-DHB is a specific inhibitor of collagen synthesis. The Col1 a1, Col1 a2, Ddr2, p38, OPN, BSP, OCN and ALP gene and protein expressions were determined using RT-qPCR and western blot. The mineralized nodules were stained using ARS.

**Fig. 3:**
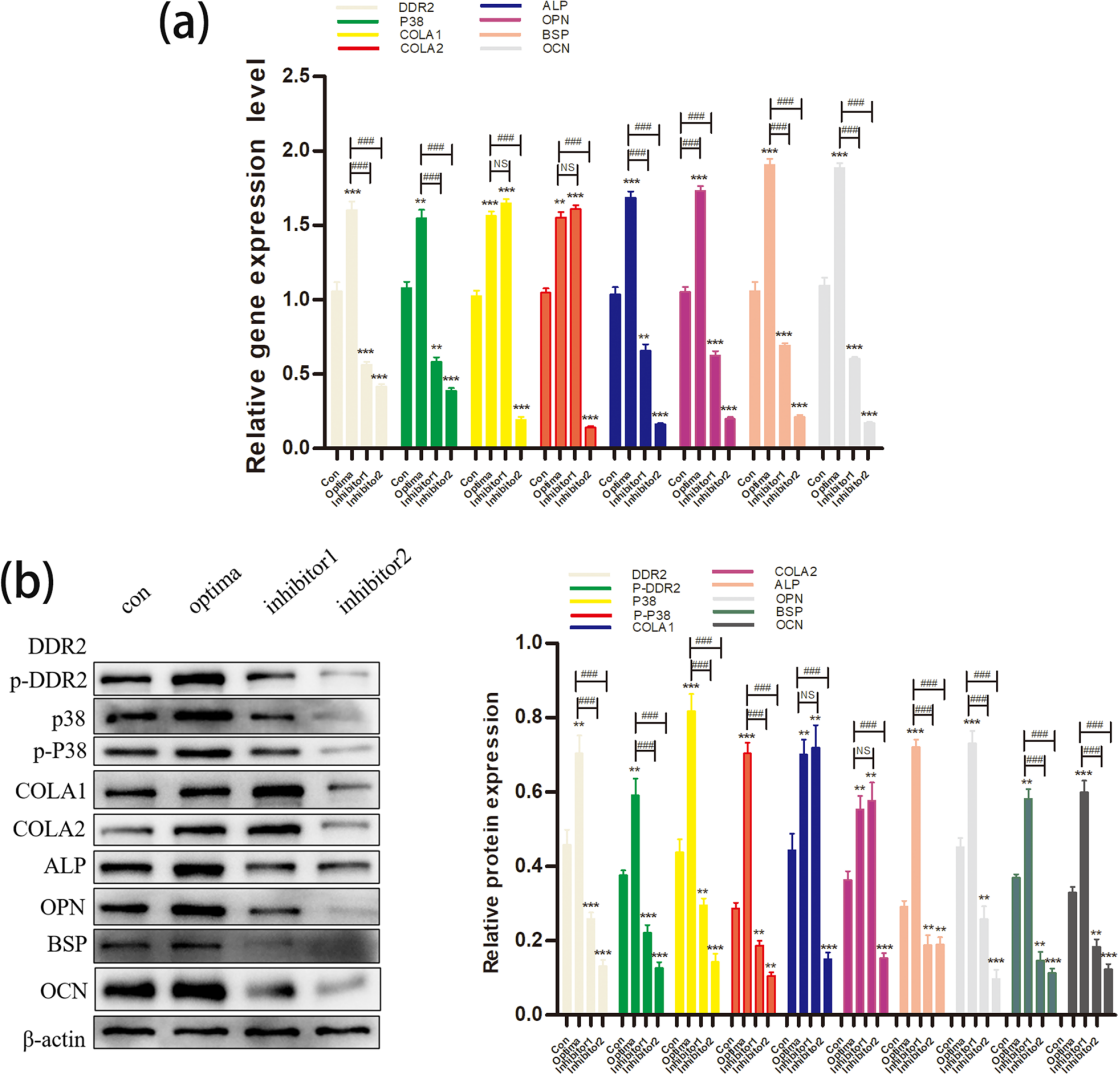
The inhibitor of collagen synthesis and the inhibitory concentration ON down-regulated the gene and protein expressions of DDR2, p38, OPN, BSP, OCN and ALP. (a) the mRNA expressions of Col1 a1, Col1 a2, DDR2, p38, OPN, BSP, OCN and ALP quantified by RT‐qPCR in response to the treatment of Con, Optima, Inhibitor1, and Inhibitor 2 group; (b) the gray value of protein bands and protein levels of Col1 a1, Col1 a2, DDR2, P-DDR2, p38, P-P38, OPN, BSP, OCN and ALP quantified by western blot analysis in response to the treatment of of Con, Optima, Inhibitor1, and Inhibitor 2 group;; *P < .05, **P < .01, ***P < .001 versus the Con group; #*P* < .05, ##*P* < .01, ###*P* < .001 versus the Optima group; the experiments were conducted in triplicates; data were expressed by means ± standard deviation (SD). ON/SPARC, osteonectin/secreted protein acidic and rich incysteine; COL1: collagen 1; DDR2: the disk protein domain receptor 2; P-DDR2: phosphorylated DDR2; P38: p38 mitogen‐activated protein kinase; P-P38: phosphorylated p38; OPN, osteopontin; BSP, bone sialoprotein; OCN, osteocalcin; RT‐qPCR: reverse transfection quantitative polymerase chain reaction; CON group: adding mineralized solution; Optima group: adding mineralized solution + 1ug/ml SPARC; Inhibitor1 group: adding mineralized solution + 100ug/ml SPARC; Inhibitor2 group: adding mineralized solution + 1ug/ml SPARC + 0.4 Mm ethyl-3,4-dihydroxybenzoate (DHB) (a specific inhibitor of collagen synthesis)

Compared with the Control group, the OPN, BSP, OCN, ALP, Ddr2 and P38 gene and protein expressions were both observably increased in the Optima group (mRNA: OPN/BSP/OCN/ALP/Ddr2, *P* < 0.001; P38, *P* < 0.01. protein: DDR2/P-DDR2/BSP, *P* < 0.01; P38/P-P38/ALP/ OPN/OCN, *P* < 0.001), and were obviously decreased in inhibitor1 and inhibitor2 group (all, *P* < 0.01). Furthermore, the expressions of Col1 a1 and Col1 a2 genes and proteins were significantly increased in Optima group and inhibitor 1 group (versus the Con group: all, *P* < 0.01), but were significantly decreased in inhibitor 2 group (versus the Con group: all, *P* < 0.01) (Fig. 3).

**Fig. 4:**
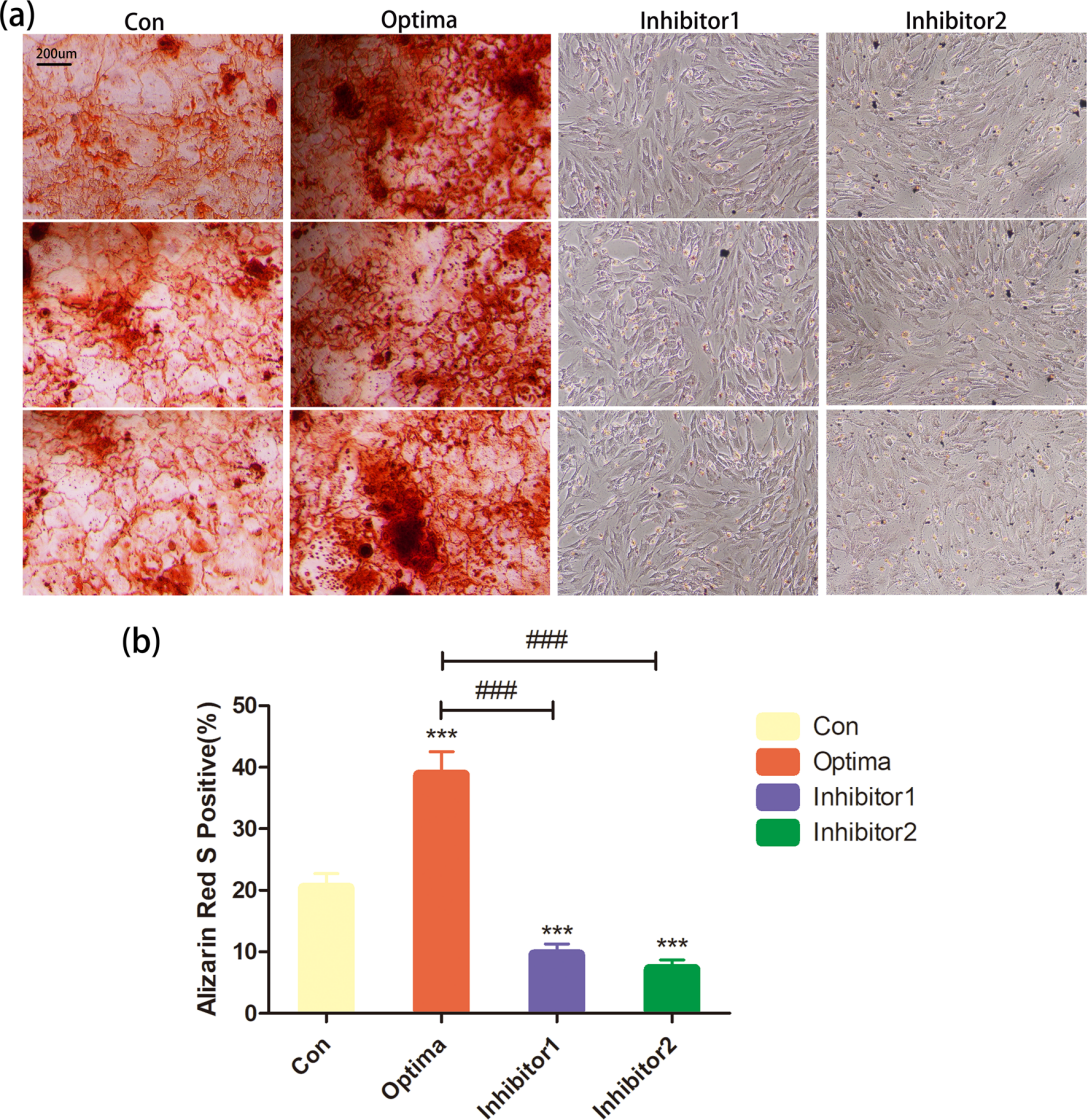
The inhibitor of collagen synthesis and the inhibitory concentration ON reduced the formation of mineralized nodules. **a** Calcium nodules observation stained with alizarin red under a microscope (× 100) after induced mineralization for 14 days in response to the treatment of Con, Optima, Inhibitor1 and Inhibitor2 group; **b** quantitative analysis of calcium nodules in each group. Bars, **a** 200 μm, *P < .05, **P < .01, ***P < .001 versus the Con group; #p < .05, ##p < .01, ###p < .001 versus the Optima group; three samples in each group were observed; data were expressed by means ± standard deviation (SD); Con group: adding mineralized solution; Optima group: adding mineralized solution + 1ug/ml SPARC; Inhibitor1 group: adding mineralized solution + 100ug/ml SPARC; Inhibitor2 group: adding mineralized solution + 1ug/ml SPARC + 0.4Mm ethyl-3,4-dihydroxybenzoate (DHB) (a specific inhibitor of collagen synthesis)

The area of mineralized nodules in the Optima group was significantly larger than that in the control group (*P* < 0.001), while in the inhibitor1 and inhibitor2 group were significantly smaller than that in the control group (*P* < 0.001) (Fig. 4).

## Discussion

Collagen and non-collagen proteins secreted by osteoblasts have a direct effect on extracellular matrix mineralization. Collagen provides the framework for mineralization, while non-collagen regulates the direction, size, and progress of crystal nucleation [19]. ON can promote collagen synthesis and conformational changes to initiate and promote mineralization [20]. However, it has also been reported that ON has a negative regulatory effect. The charge and orientation of the acid residues (glutamate and l-L-Aspartic Acid) of ON can interfere with HA formation [15, 16]. In order to investigate whether there is a dose-dependent effect of ON on osteoblast mineralization, the groups were divided according to the different concentrations of ON in the first stage of this experiment. The results showed that 1 ug/ml ON significantly increased osteoblast mineralization, which was consistent with our previous results [17], whereas 100 ug/ml ON significantly inhibited osteoblast mineralization. Related studies have provided similar results. 0.1–0.3 ug/ml of ON can obviously promote the formation of collagen fiber bundles and accelerate the mineralization of collagen [21], while at a concentration of 100 ug/ml, it will lose its nucleation activity [13]. The above results confirmed that ON has a two-way dose-dependent regulation on osteoblast mineralization.

ON and P38 have extensive correlation in cell growth and metabolism. The overexpression of ON-activated p38 MAPK-HSP27 signal pathway is the current hotspot in the diagnosis and treatment of malignant tumors [22–24]. The mechanism of ON-activated p38 MAPK pathway has also been widely demonstrated in the research of promoting Endothelium proliferation, corneal epithelial cell differentiation, and ectopic ossification [25–27]. Our previous studies have also confirmed that ON regulates osteoblast mineralization through the P38 pathway [17]. The discoid domain receptor, DDR, is a tyrosine kinase receptor that is widely expressed on the cell surface and can be activated slowly and continuously by triple-helix collagen, which is essential for bone development and regeneration [28, 29]. ON has the functional and structural basis of activating the binding of collagen to DDR2, which can promote collagen synthesis and induce collagen conformational changes. Genetic studies provide evidence of the importance of DDR2 in bone development. Mutations or deletions of DDR2 can lead to developmental deformities or severe defects in bone formation [30–32]. Activation of the DDR receptor is involved in the regulation of biological behavior between cells and ECM, involving the p38 MAPK pathway. Activation of DDR-2 receptor can induce pathological changes in chondrocytes, stimulate osteoblast differentiation and bone formation through p38 MAPK pathway [33, 34]. The mechanism by which collagen-binding DDR2 activates p38 MAPK pathway, a downstream of ERK/MAP kinase proliferation, is thought to be an essential signaling pathway required for normal skeletal development [35, 36]. Our previous experiments also confirmed that collagen-DDR2-activated P38 pathway was an important mechanism for ON positively regulating osteoblast mineralization [18]. In order to confirm whether this pathway was also affected in the Inhibitory concentration group, genetic detection of collagen, Ddr2 and P38 was added in the second stage. The results showed that the expression of Ddr2-P38 pathway was indeed significantly inhibited in the Inhibitory concentration group.

Type I collagen is the main protein of bone matrix and serves as a template in the process of apatite mineralization [19]. Although the Ddr2-P38 pathway was inhibited in the Inhibitory concentration ON group, collagen synthesis was still significantly increased. 3,4-DHB is a competitive prolyl hydroxylase inhibitor, which can specifically block collagen synthesis [37]. To further confirm the role of collagen in ON regulation of osteoblast mineralization, 3,4-DHB was added to the Optima concentration of ON in the third stage of the experiment. The results showed that the synthesis of type 1 collagen in the Inhibitor2 group (added with 3,4DHB) was significantly reduced, and the positive regulation of Optima ON on mineralization was significantly inhibited, which suggested that collagen was still a key factor in the regulation of osteoblast mineralization by ON. However, the reason why collagen synthesis was increased but mineralization was inhibited in the Inhibitory concentration ON group remains to be further explored. Osteoblast mineralization is a complex physiological process involving multiple cytokines and different pathways. Although collagen synthesis is increased, ON has a strong ability to bind collagen, and its premature excessive binding to collagen may instead be a potential reason for inhibiting mineralization. In addition, DDR2 and ON have the same GVMGFO motif and a common pattern of collagen recognition [38], excessive ON may also compete with DDR2 to bind collagen, thus preventing DDR2 from activating P38. Of course, the exact mechanism needs further experimental confirmation.

In conclusion, this study confirmed that ON had a bidirectional dose-dependent regulatory effect on osteoblast mineralization, and the activation of P38 pathway by collagen binding to DDR2 was also an important molecular mechanism. This study provides a new target for precisely regulating bone matrix mineralization, and may provide effective means and objective experimental basis for future animal experiments and clinical application of bone repair and reconstruction.

## Data Availability

We declared that materials described in the manuscript, including all relevant raw data, will be freely available to any scientist wishing to use them for non‑commercial purposes, without breaching participant confidentiality.
